# Development and validity of type II sulcus vocalis in excised canine larynx

**DOI:** 10.1002/wjo2.193

**Published:** 2024-06-09

**Authors:** Ting Gong, Peng‐Cheng Yu, Chao Xue, An‐Rong Sun, Yi Zhang, Rui Fang, Bing‐Hua Zhu

**Affiliations:** ^1^ School of Rehabilitation Science Shanghai University of Traditional Chinese Medicine Shanghai China; ^2^ Institute of Rehabilitation Medicine Shanghai Academy of Traditional Chinese Medicine Shanghai China; ^3^ Engineering Research Center of Traditional Chinese Medicine Intelligent Rehabilitation Ministry of Education Shanghai China; ^4^ ENT institute and Department of Otorhinolaryngology, Eye & ENT Hospital Fudan University Shanghai China; ^5^ Department of Emergency & Intensive Care Unit Shuguang Hospital, Affiliated to Shanghai University of Traditional Chinese Medicine Shanghai China

**Keywords:** animal model, canine, excised larynx, sulcus vocalis, vocal folds

## Abstract

**Objectives:**

This study aims to develop a sulcus vocalis model in the excised canine larynx and to investigate the validity of the model.

**Methods:**

Type II sulcus vocalis was created by continuous intradermal suture in six excised canine larynges. We investigated the validity of the model based on phonatory (aerodynamic and acoustic) measures, laryngeal videostroboscopy (LVS), and histological examination. The aeroacoustic parameters included phonation threshold pressure (PTP), fundamental frequency (*F*_o_), jitter %, shimmer %, and harmonics‐to‐noise ratio (HNR).

**Results:**

In all the experimental specimens, there were significant increases in the PTP ([6.61 ± 1.66] cm H_2_O, *p* < 0.01), the *F*_o_ ([106.48 ± 49.59] Hz, *p* = 0.003), the jitter ([0.76 ± 0.42]%, *p* = 0.007), the shimmer ([6.87 ± 2.99%, *p* = 0.002]), while the HNR decreased ([5.71 ± 4.68] dB, *p* = 0.031), compared to that of the untreated canine larynges. The estabilished model simulated the histology with type Ⅱ sulcus.

**Conclusions:**

Type Ⅱ sulcus vocalis was successfully created in excised canine larynx. The aeroacoustic and LVS analysis of this model resembled the characteristics of patients with sulcus vocalis. The model could be helpful to elucidate the pathology of the phonation, and evaluate and compare the treatments for sulcus vocalis.

## Key points

- Significant findings of the study: This study established a type II sulcus vocalis model in excised canine larynx.
- What this study adds: This study provided an experimental model to elucidate the pathology of the phonation, evaluate and compare the treatments for sulcus vocalis.

## INTRODUCTION

Sulcus vocalis is used to describe a groove of varied length and depth parallel to the free edge of the vocal fold (VF). It is a kind of VF malformation with epithelium presenting in the lamina propria (LP) or deeper due to the reduced or lost LP.^1^ It can be congenital or acquired. The most commonly used classification of sulcus vocals is proposed by Ford et al.^2^ based on its severity: A type I sulcus, commonly referred to as a “physiologic sulcus,” is a depression of the epithelium into only the superficial lamina propria (SLP), which is thought to have no functional impact and not considered pathologic; A type II sulcus, referred to as a “sulcus vergeture,” is characterized by loss of the SLP with extension to the vocal ligament; A type Ⅲ sulcus is often referred to as a “pit” or “pouch” because it is a focal indentation that extends into the vocal ligament or deeper. Patients present with a variable degree of hoarseness, associated with vocal fatigue, breathiness, and vocal strain, due to glottic incompetence, poor VF vibratory function, and subsequent excessive ventricular fold adduction. Sulcus vocalis is not uncommon in clinical practice and is easily overlooked when combined with other pathological changes of VFs. It was reported a total of 61.7% of the patients with benign VF lesions had bilateral sulcus vocalis.^3^ The prevalence of sulcus vocalis in patients without vocal complaints was recently found to be 16.3%.^4^

The diagnosis and treatment of sulcus vocalis can be both challenging and rewarding. Patients with sulcus are hard to diagnose by indirect laryngoscopy, fibroscopy as well as stroboscopy. The gold standard diagnostic method is the palpation of the VFs under microlaryngoscopy with a high magnification. A prospective study showed only 34% of patients were diagnosed by stroboscopy, further confirmed after undergoing subsequent suspension microlaryngoscopy.^5^ Laryngeal videostroboscopy (LVS) findings of pathologic sulcus cases had revealed that the length and depth of sulcus configuration could affect the vibration amplitude, mucosal wave propagation, and glottal closure.^6^ It was reported the observed decreased maximum phonation time, decreased fundamental frequency range, and increased airflow during phonation in sulcus vocalis.^7^ Patients with sulcus vocalis can be candidates for surgical interventions when voice therapy is proved ineffective and functional limitations persist.^6^ The surgical treatment is to loosen and release the tissue adhesion at the bottom of the sulcus vocalis with or without medialization therapy to reduce the glottic leakage.^3^ Limited studies referring to the surgical treatment are available and their results differ. In some cases, the quality of patients' voices may be worse postsurgery.^8^ It is urgent to develop the sulcus vocalis model to explore the underlying pathophysiology and seek for effective treatment.

## METHODS

### Larynx preparation

Eight larynges with similar size were harvested from canines killed for nonresearch purpose. Following excision, the larynges were frozen in 0.9% saline solution. Six larynges were used for phonatory measures and two for the histological examination. In preparation, each larynx was slow‐thawed in a 37°C bath to reintroduce pliability. The epiglottis, cuneiform cartilages, corniculate cartilages, and ventricular folds as well as the superior cornu were dissected away to expose the true VFs. A segment of the trachea was preserved and sealed off by the inflated balloon of the endotracheal tube. Compressed air was provided by a ventilator (ResMed AutoSet II) and passed through an airflow adjust controller and a flowmeter (MF5706‐N‐10; Siargo Ltd.) which was connected to the endotracheal tube. VF adduction was achieved by 5‐0 polypropylene sutures placed through the vocal process and arytenoid cartilage.

### Sulcus vocalis model

**Figure 1:**
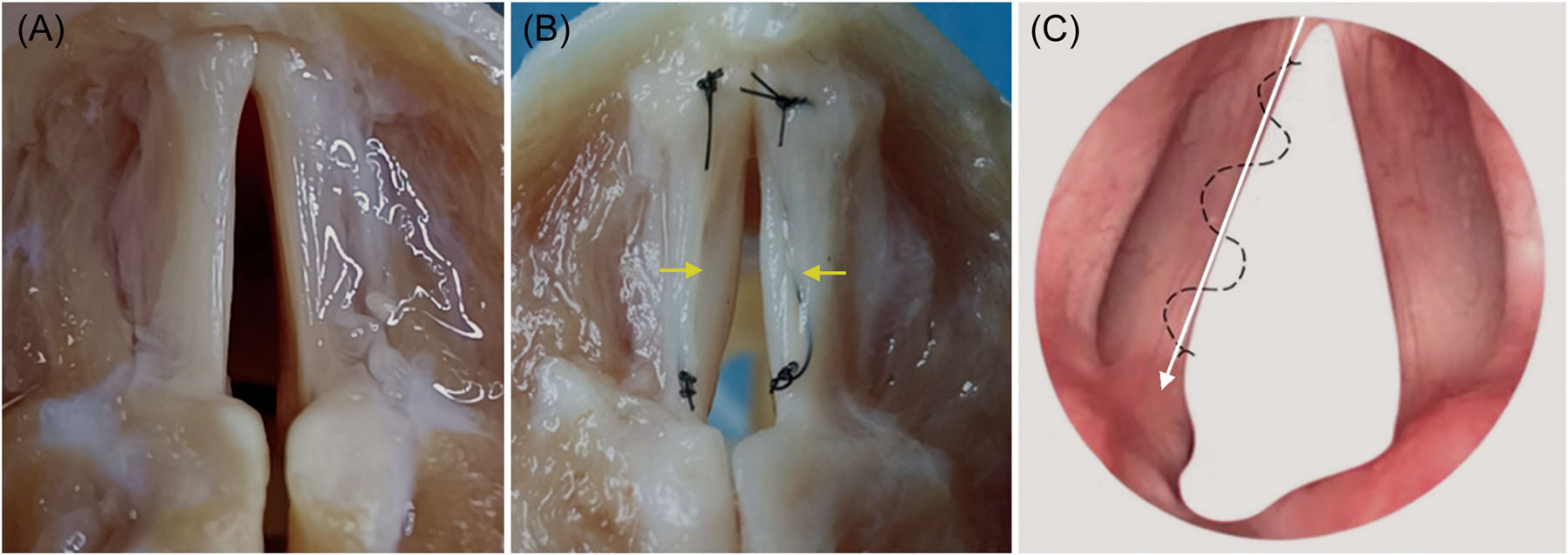
Sulcus vocalis model. (A) The untreated excised canine larynx. (B) The established sulcus vocalis marked with the yellow arrows in excised canine larynx. (C) Illustration of the continuous intradermal suture on vocal fold.

The type II sulcus vocalis was created by continuous intradermal sutures of 6‐0 polypropylene (Figure 1A). The suture method on VF was illustrated in Figure 1C. The epithelium was invaginated into the vocal ligament along the long axis of the VF. All suture was performed by an experienced expert to ensure consistency.

### Experimental procedures

The phonatory measurements and LVS were carried out for each specimen twice, before and after the suture. Phonation threshold pressure (PTP) was measured by a barometer when a clear and pronounced tone was reached. The premodeling and postmodeling acoustic measurements were collected by a digital voice recorder (Newsmy RV28) positioned at an approximate distance of 15 cm from the larynx. The most stable 1.0‐s portion of the acoustic waveform was selected and measured with Praat software. The *F*_o_, HNR, jitter, and shimmer were obtained.

LVS tests were performed by the operating surgeon—laryngologist—phoniatrist (BM) with a stroboscopy (Karl Storz 20140020) and 70° rigid laryngoscope (Karl Storz 8706CA) and the results were assessed by two separate examiners. The specific laryngeal functions were evaluated by various scales (each score 0‐3): glottal closure (0, complete; 1, small gap; 2, moderate gap; 3, large gap); amplitude (0, normal; 1, mildly diminished; 2, moderately diminished; 3, severely diminished); and mucosal wave (0, normal; 1, mildly restricted; 2, moderately restricted; 3, completely lacking).

### Histological examination

The larynges were embedded in paraffin blocks and then sectioned at 4‐μm thick along the coronal axis of the larynx. Next, slides were stained with hematoxylin and eosin (water washing, differentiation, water washing, eosin alcohol staining solution), dehydrated, transparent, and mounted with neutral gum.

### Statistical analysis

SPSS 20.0 (IBM Corp.) was used for the statistical analysis. The paired sample *t*‐tests were performed to compare the means of the parameters. Results were expressed as mean ± SD. The significance level for all analyses was defined as *p* < 0.05.

## RESULTS

Phonatory characteristics of six larynges before and after suture were presented in Table 1. The average PTP was (7.57 ± 1.21) cm H_2_O and (14.19 ± 2.67) cm H_2_O (*d* = 6.61 ± 1.66, *p* < 0.001). The average *F*_o_ was (200.04 ± 39.33) Hz and (306.52 ± 56.23) Hz (*d* = 106.48 ± 49.59, *p* = 0.003). The average jitter was (0.19 ± 0.06)% and (0.96 ± 0.44)% (*d* = 0.76 ± 0.42, *p* = 0.007*)*. The average shimmer was (7.77 ± 2.54)% and (14.64 ± 1.05)% (*d* = 6.87 ± 2.99, *p* = 0.002). The average HNR was (13.62 ± 2.61) dB and (7.92 ± 3.49) dB (*d* = −5.71 ± 4.68, *p* = 0.031).

**Table 1:** Acoustic and aerodynamic parameters before and after the buried suture (*n* = 6, mean ± SD).

Variables	Pre‐SV (a)	Post‐ SV (b)	d (b‐a)	*p* Value
PTP, cm H_2_O	7.57 ± 1.21	14.19 ± 2.67	6.61 ± 1.66	<0.01**
*F*_0_, Hz	200.04 ± 39.33	306.52 ± 56.23	106.48 ± 49.59	0.003**
Jitter, %	0.19 ± 0.06	0.96 ± 0.44	0.76 ± 0.42	0.007**
Shimmer, %	7.77 ± 2.54	14.64 ± 1.05	6.87 ± 2.99	0.002**
HNR, dB	13.62 ± 2.61	7.92 ± 3.49	−5.71 ± 4.68	0.031*

Abbreviations: *F*_0_, fundamental frequency; HNR, harmonics‐to‐noise ratio; PTP, phonation threshold pressure; SV, sulcus vocalis.

* *p* < 0.05

** *p* < 0.01.

LVS scores were presented in Table 2. In the rows, the state of glottal closure, amplitude, and mucosal wave was premodeling, meaning the state before suturing. In the columns, we can see the postmodeling change of their state in terms of the assessment of LVS patterns. The condition of the glottal gap was significantly worse after the modeling. In addition, amplitude and mucosal wave values were significantly diminished and restricted postmodeling.

**Table 2:** Comparison of preassessment and postassessment of LVS patterns (Glottal closure, Amplitude, Mucosal wave).

Glottal closure	Complete (post)	Small gap (post)	Moderate gap (post)	Large gap (post)
Complete (pre)	0	1	2	1
Small gap (pre)	0	0	1	1
Amplitude	Normal (post)	Mildly diminished (post)	Moderately diminished (post)	Severely diminished (post)
Normal (pre)	0	0	1	3
Mildly diminished (pre)	0	0	0	2
Mucosal wave	Normal (post)	Mildly restricted (post)	Moderately restricted (post)	Severely restricted (post)
Normal (pre)	0	0	1	3
Mildly restricted (pre)	0	0	0	2

*Note*: Row: premodeling; Column: postmodeling.

Abbreviation: LVS, laryngeal videostroboscopy.

**Figure 2:**
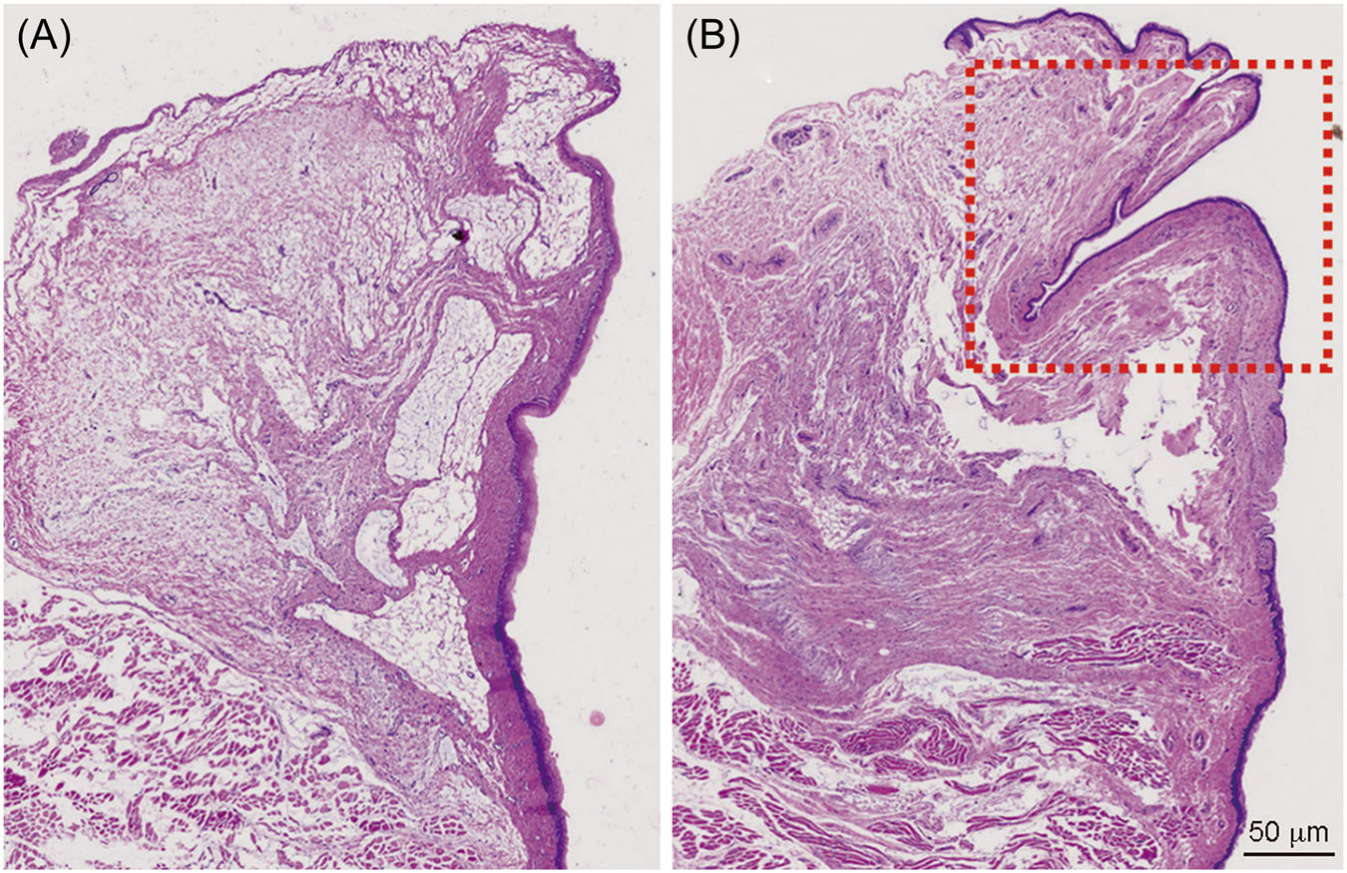
Histological determination of the vocal fold. At ×20 magnification. Scale bars are 500 μm. (A) The untreated larynx was presented as the ordered arrangement. (B) The typeⅡ sulcus vocalis model marked with the red dashed box was characterized by the loss of the superficial lamina propria (SLP) with extension to the vocal ligament.

The morphology of type II sulcus vocalis model in excised canine larynx was presented in Figure 1A. Histological results were shown in Figure 2. The type II sulcus vocalis model was characterized by the loss of the SLP with extension to the vocal ligament (Figure 2A). The untreated larynx was presented as the ordered arrangement (Figure 2B).

## DISCUSSION

Sulcus vocalis is the pathophysiologic changes of the VF. The reduced or lost lamina propira causes a “spindle‐shaped glottis” with incomplete glottal closure and the deterioration of VF pliability. Few studies had reported LVS and aeroacoustic measurements of sulcus vocalis, and the limited results before and post surgery were diversified. Most patients had been treated with VF injection and these treatment options were made according to the width of the glottal gap during phonation under LVS. A study reported an experimental animal model for sulcus vergeture in rabbits, however, it was short of the phonatory measures.^9^ In this study, the significantly diminished amplitude and mucosal wave were observed in sulcus vocalis models, and there was a moderate to large glottal gap. The increased stiffness and the decreased mass of the VFs caused by the reduction or loss of the LP may result in an inappropriately high‐pitched voice. The habitual vocal pitch is an important factor of the vocal quality, which usually makes someone's voice unique. It depends on gender, age as well as the airway anatomy. The inappropriately high‐pitched voice can be perceived as feminine or childlike when patients talk on the telephone or communicate in a nonvisual manner. However, for these patients with other VF lesions combined, the increased mass of the VFs may result in the decreased fundamental frequency.^3^ In our study, with the increased stiffness of the VFs in these sulcus vocalis models, the PTP elevation was (6.61 ± 1.66) cm H_2_O, and the *F*_o_ elevation was (106.48 ± 49.59) Hz. The jitter and shimmer elevations were (0.76 ± 0.42)%, *p* = 0.007 and (6.87 ± 2.99)%, *p* = 0.002 respectively. The HNR downregulation was 5.71 ± 4.68 (*p* = 0.031). Our results were consistent with the changes of aeroacoustic parameters, LVS results, and histology in vivo. It may be promising for further use in the excised larynx to investigate the phonation mechanisms and the evaluation of sulcus vocalis.

Voice therapy can be effective in treating mild sulcus vocalis, such as type I, but be little effective for patients with severe sulcus vocalis, such as type II. Surgical interventions for sulcus vocalis include medialization injection laryngoplasty, undermining the sulcus, and medialization thyroplasty.^10^ However, studies had reported that the surgical outcomes were unpredictable. More conservative surgical techniques and the recent incorporation of angiolytic lasers to treat sulcus vocalis have emerged as promising treatments in a less aggressive way. Kocak et al.^11^ reported a less invasive modification of the standard Isshiki technique that provided better stability and ease of adjustability. The premodeling *ƒ*_o_ of sulcus vocalis was 205.71 Hz, and it decreased to 146.43 Hz postsurgery. However, instability is still a potential defect of this technique. It can interfere with the fine adjustments made during surgery and result in postoperative complications related to displacement. Lee et al.^12^ revealed that male patients receiving the angiolytic laser glottoplasty with the adjuvant injection laryngoplasty group showed a greater decrease in *F*_o_, compared to those who received the angiolytic laser glottoplasty alone. Their results suggested that adjuvant injection laryngoplasty might be beneficial for patients who had the abnormally elevated *F*_o_ preoperatively, particularly male patients. Our sulcus vocalis models can be promising for further use in the excised larynx to explore the optimized surgical intervention.

This study is limited by the small sample size. Further studies should be performed with increased sample sizes. In addition, the current pathophysiologic process leading to sulcus vocalis is not well established. The pathological conditions in sulcus vocalis models in excised larynx may be different from those in vivo. The sulcus vocalis in vivo involves more than just loss of SLP volume but also sparsity of vocal ligament, loss of muscular volume, and dysfunction of macula flava. This model does not take these into account and as such is limited. These limitations can be the direction of effort for future studies.

## AUTHOR CONTRIBUTIONS

Ting Gong contributed to the study design, conception, and drafting the manuscript. Pengcheng Yu, Chao Xue, and Anrong Sun contributed to the project administration, data collection, and data analysis. Rui Fang and Binghua Zhu contributed to the supervision and approval of the final manuscript.

## CONFLICT OF INTEREST STATEMENT

The authors declare no conflict of interest.

## ETHICS STATEMENT

All experimental procedures about animals and their care were approved by the institutional review board of the Eye, Ear, Nose, and Throat Hospital of Fudan University. The above manuscript is the authors' own original work, which has not been previously published and is not in submission elsewhere. The paper reflects the authors' own research and analysis in a truthful and complete manner and properly credits the meaningful contributions of coauthors and coresearchers.

## Data Availability

Data are available on request from the authors.
